# A Novel Discretization Procedure in the CSI-FEM Algorithm for Brain Stroke Microwave Imaging

**DOI:** 10.3390/s23010011

**Published:** 2022-12-20

**Authors:** Valeria Mariano, Jorge A. Tobon Vasquez, Francesca Vipiana

**Affiliations:** Department of Electronics and Telecommunications, Politecnico di Torino, 10129 Torino, Italy

**Keywords:** contrast source inversion method, finite element method, brain stroke, microwave imaging

## Abstract

In this work, the contrast source inversion method is combined with a finite element method to solve microwave imaging problems. The paper’s major contribution is the development of a novel contrast source variable discretization that leads to simplify the algorithm implementation and, at the same time, to improve the accuracy of the discretized quantities. Moreover, the imaging problem is recreated in a synthetic environment, where the antennas, and their corresponding coaxial port, are modeled. The implemented algorithm is applied to reconstruct the tissues’ dielectric properties inside the head for brain stroke microwave imaging. The proposed implementation is compared with the standard one to evaluate the impact of the variables’ discretization on the algorithm’s accuracy. Furthermore, the paper shows the obtained performances with the proposed and the standard implementations of the contrast source inversion method in the same realistic 3D scenario. The exploited numerical example shows that the proposed discretization can reach a better focus on the stroke region in comparison with the standard one. However, the variation is within a limited range of permittivity values, which is reflected in similar averages.

## 1. Introduction

Microwave imaging (MWI) is a widely exploited technique in the medical field; indeed, the sensitivity of the microwave frequencies to the dielectric contrast makes this method suitable for different clinical applications. It can be used in combination with hyperthermia for cancer therapy, as in [1], or microwave measurement data can be analyzed through a machine learning algorithm to classify the brain stroke’s presence and typology [2]. In [3,4,5], MWI is employed for the diagnosis of breast cancer, in [6] for the detection of traumatic intracranial hemorrhage, and in [7] for brain stroke detection. In this work, MWI is exploited for reconstructing the dielectric properties distribution of the head tissues for brain stroke imaging.

The basic idea is to determine some information about an unknown scatterer in the domain of interest (DoI) from its scattered field through the solution of an inverse ill-posed problem. This kind of problem is very demanding, and some techniques, such as the Tikhonov regularization, can be used to solve them, as described in [8].

In this work, we use the contrast source inversion (CSI) method [9] for the solution of the inverse problem. The CSI method is an iterative nonlinear algorithm widely exploited in MWI technology, thanks to its capability to reconstruct the dielectric properties distribution quantitatively inside the DoI. Another quantitative algorithm used in the literature for brain microwave imaging is the distorted Born iterative method, as in [10,11].

The applications of the CSI algorithm are multiple. In the food industry, e.g., [12], it is used to detect hot spots of moisture in grain bins; in [13], it is exploited to reconstruct the distribution of the electrical conductivity between boreholes in the oil industry. Moreover, the CSI algorithm is widely applied to medical issues. For example, in [14], it is combined with magnetic resonance images to detect the local absorption rate distribution. In our work, as in [15], the algorithm is exploited for reconstructing the dielectric properties distribution of the head tissues for brain stroke imaging. The CSI method can also be combined with artificial intelligence and machine/deep learning algorithms. In [16], the CSI method is enhanced through deep learning for breast cancer detection. Instead, in [17], the tomography of electrical properties is realized through a deep learning first reconstruction followed by the CSI method.

Here, the CSI algorithm uses a finite element method (FEM) solver to discretize the whole domain. FEM allows using an unstructured and nonuniform mesh, fundamental characteristics to discretize a realistic scenario accurately. In particular, we use an in-house customized 3D FEM solver [18,19], which also comprises the numerical model of the implemented antenna system described in [20]. The CSI algorithm, combined with an FEM solver, was also used for a 2D scenario in [21] and a 3D one in [15], but, in both cases, the sources were simple points or elementary dipoles. In this paper, the complete antenna model, together with its coaxial feeding, is included in the inversion algorithm to improve the incident field estimation in the DoI, as well as the scattering parameter evaluation at the antennas’ ports.

The main aim of this work is to propose novel basis functions to discretize the contrast source variables based on the vector basis functions commonly used in the 3D FEM for the fields. This novel discretization is validated through a numerical analysis that quantitatively compares it with the standard one in terms of efficiency and accuracy in the whole CSI process for brain stroke imaging in a realistic scenario.

The paper is organized as follows. In Section 2, there is a brief description of the implemented microwave imaging system and head phantom. Section 3 reports an overview of the CSI method and, in Section 4, there is a detailed description of the standard and the novel discretizations of the contrast source variables. In Section 5, both the discretizations are numerically analyzed in terms of accuracy. Then, they are applied to a brain stroke imaging problem to assess their performances using the same scenario. Finally, in Section 6, the conclusion and perspectives are summarized.

## 2. Microwave Imaging System and Head Model

**Figure 1:**
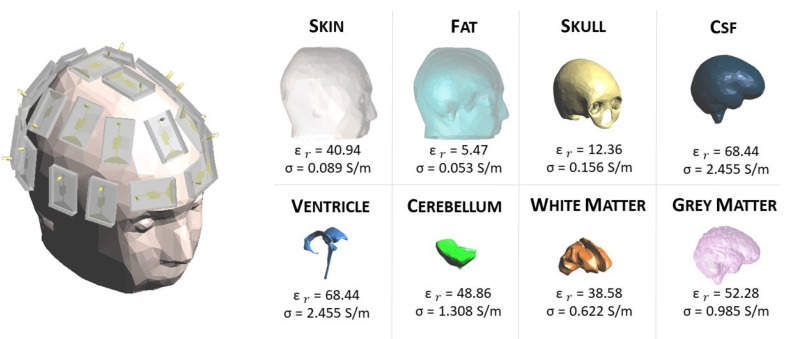
On the left: the MWI system; on the right: all the tissues embedded in the 3D anthropomorphic phantom with their dielectric properties at 1 GHz [26].

The herein MWI-exploited system comprises 24 low-weight flexible antennas acting as both transmitter and receiver (for more details, see [20]). The antennas are distributed conformally around the head to form a helmet and covered by a thin layer of coupling medium, with a relative permittivity of around 20, which facilitates the field penetration inside the head at a working frequency equal to 1 GHz. The working frequency is chosen to obtain a trade-off between the wave penetration in the head tissues and the imaging resolution that has opposite requirements on it (i.e., at high frequencies, we have high resolution but poor wave penetration, at low frequencies, it is the opposite). Moreover, as detailed in [22], there is a frequency band corresponding to around 1.5–4 GHz, in which the transmission coefficient is significantly reduced due to the head dielectric tissues’ multilayer configuration. Hence, the suggested frequency choice is below 1.5 GHz because, at frequencies higher than 4 GHz, the tissues’ losses are much higher, and the field cannot penetrate through the head. This version of the MWI system is more compact in comparison with the previous one proposed in [23]. It allows to reach the portability requirement for the final system prototype, as shown in Figure 1. The FEM, used in combination with the CSI algorithm, comprises the modeling of the entire antenna system and coaxial feeding, allowing an accurate estimation of the field distribution in the DoI and at the antennas’ ports.

In this work, a numerical 3D anthropomorphic phantom is exploited. In particular, the phantom belongs to the National Library of Medicine within the Visible Human Project [24]. This project contains computerized tomography and magnetic resonance images of the cross-sectional cryosection of a human man and a human female body. The data can be downloaded from the website [25]. All the tissues involved in the healthy reference human head are shown in Figure 1 with the relative permittivity, $\epsilon_{r}$, and conductivity, σ, at 1 GHz [26].

## 3. Contrast Source Inversion Method

In this section, we briefly summarize the CSI method to set up the notation for the following description of the proposed discretization of the contrast source variables.

The CSI algorithm is based on the definition of two main variables. The first one is the dielectric contrast, $\chi (\underline{r})$, between the relative complex permittivity of the background (reference) scenario, $\epsilon_{b}\left(\underline{r}\right)$, and of the scenario under test, ${\tilde{\epsilon }}_{r}\left(\underline{r}\right)$:

(1) $$\chi \left(\underline{r}\right)\overset{\Delta }{=}\frac{{\tilde{\epsilon }}_{r}\left(\underline{r}\right)-\epsilon_{b}\left(\underline{r}\right)}{\epsilon_{b}\left(\underline{r}\right)}.$$

where $\underline{r}$ indicates a generic point in the DoI.

The second variable is the contrast source, ${\underline{\omega }}_{t}\left(\underline{r}\right)$, which identifies equivalent sources in the DoI associated with the transmit antenna *t*. The contrast source causes the (scattered) field received at the antennas’ ports in the scenario under test. They are also called induced, passive or secondary sources and are defined with the so-called object equation:

(2) $${\underline{\omega }}_{t}\left(\underline{r}\right)\overset{\Delta }{=}\chi \left(\underline{r}\right){\underline{E}}_{t}^{\mathrm{tot}}\left(\underline{r}\right),$$

where ${\underline{E}}_{t}^{\mathrm{tot}}\left(\underline{r}\right)$ is the total field radiated by the antenna *t* in the DoI of the scenario under test (scenario with the target). On the contrary, the same quantity in the background scenario (scenario without the target) is called incident field, ${\underline{E}}_{t}^{\mathrm{inc}}\left(\underline{r}\right)$. The evaluation of the scattered field, ${\underline{E}}_{t}^{\mathrm{sct}}\left(\underline{r}\right)$, is simply the difference between the total and the incident field as:

(3) $${\underline{E}}_{t}^{\mathrm{sct}}\left(\underline{r}\right)={\underline{E}}_{t}^{\mathrm{tot}}\left(\underline{r}\right)-{\underline{E}}_{t}^{\mathrm{inc}}\left(\underline{r}\right).$$

For each transmit antenna *t*, the ${\underline{E}}_{t}^{\mathrm{sct}}\left(\underline{r}\right)$ and the ${\underline{\omega }}_{t}\left(\underline{r}\right)$ are linked together via the wave equation:

(4) $$\nabla \times \nabla \times {\underline{E}}_{t}^{\mathrm{sct}}\left(\underline{r}\right)-k_{b}^{2}\left(\underline{r}\right){\underline{E}}_{t}^{\mathrm{sct}}\left(\underline{r}\right)=k_{b}^{2}\left(\underline{r}\right){\underline{\omega }}_{t}\left(\underline{r}\right),$$

where the background medium wave number is identified with $k_{b}\left(\underline{r}\right)=\omega \sqrt{\mu_{0}\epsilon_{0}\epsilon_{b}\left(\underline{r}\right)}$, in which ω is the angular frequency, $\mu_{0}$ is the free space permeability, and $\epsilon_{0}$ is the free space permittivity.

In the CSI method, $\chi (\underline{r})$ and ${\underline{\omega }}_{t}\left(\underline{r}\right)$ are updated at each iteration *n*, so that a cost functional, $F^{\mathrm{CSI}}$, is minimized. The cost functional asses the error between known data, i.e., the scattered field at the antennas’ ports and the incident field in the DoI due to the transmit antenna *t*, and the corresponding ones obtained by the algorithm procedure. The $F^{\mathrm{CSI}}$, at the iteration *n*, can be written as:

(5) $$F^{\mathrm{CSI}}\left\{\chi_{n}\left(\underline{r}\right),{\underline{\omega }}_{t,n}\left(\underline{r}\right)\right\}=F^{S}\left\{{\underline{\omega }}_{t,n}\left(\underline{r}\right)\right\}+F^{D}\left\{\chi_{n}\left(\underline{r}\right),{\underline{\omega }}_{t,n}\left(\underline{r}\right)\right\}$$

where

(6) $$F^{S}\{{\underline{\omega }}_{t,n}\left(\underline{r}\right)\}=\frac{\sum_{t=1}^{T}{\left\|{\underline{E}}_{t}^{\mathrm{sct}}\left(\underline{r}\right)-G_{S}\left\{{\underline{\omega }}_{t,n}\left(\underline{r}\right)\right\}\right\|}_{S}^{2}}{\sum_{t}{\left\|{\underline{E}}_{t}^{\mathrm{sct}}\left(\underline{r}\right)\right\|}_{S}^{2}},$$

(7) $$F^{D}\{\chi_{n}\left(\underline{r}\right),{\underline{\omega }}_{t,n}\left(\underline{r}\right)=\frac{\sum_{t=1}^{T}{\left\|\chi_{n}\left(\underline{r}\right){\underline{E}}_{t}^{\mathrm{inc}}\left(\underline{r}\right)-{\underline{\omega }}_{t,n}\left(\underline{r}\right)+\chi_{n}\left(\underline{r}\right)G_{D}\left\{{\underline{\omega }}_{t,n}\left(\underline{r}\right)\right\}\right\|}_{D}^{2}}{\sum_{t}{\left\|\chi_{n}\left(\underline{r}\right){\underline{E}}_{t}^{\mathrm{inc}}\left(\underline{r}\right)\right\|}_{D}^{2}}.$$

**Figure 2:**
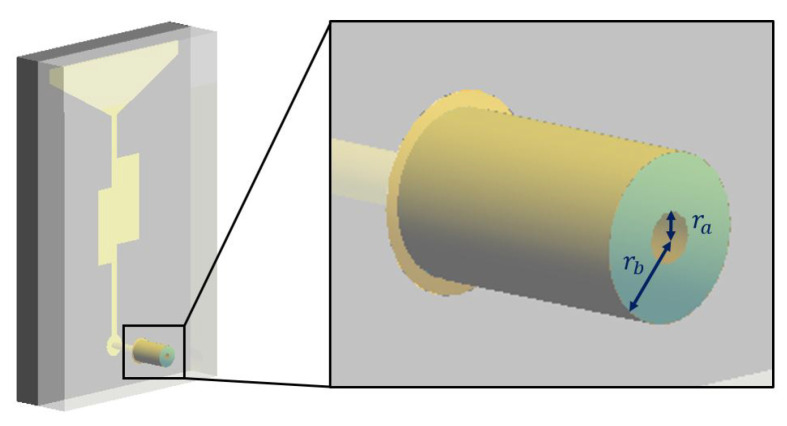
Whole model of antenna with closeup on the coaxial cable port.

The functionals $F^{S}$ and $F^{D}$ evaluate the mismatch between the known quantities and those estimated at iteration *n* in the domains S, where the antennas are located (i.e., at the antennas’ ports), and D, which corresponds to the DoI, respectively. Moreover, *T* is the total number of antennas, ${∥\cdot ∥}^{2}$ identifies the Euclidean norm, and $G_{S}$ and $G_{D}$ are operators that return the scattered field values in the domain S and D, respectively [15]. In the cost functional described above, a regularization term can also be included to speed up the algorithm’s convergence, as described in [27]. In this work, the CSI algorithm was modified to link the scattering parameters at the coaxial antennas’ ports to the corresponding scattered field in *S*, as requested in (6). A generic scattering parameter $S_{m,t}$ is written by definition as:

(8) $$S_{m,t}={\left.\frac{b_{m}}{a_{t}}\right|}_{a_{k}=0,k\neq t}$$

where the antenna *t* is the transmitter and the antenna *m* the receiver, and $a_{t}$ and $b_{m}$ are the incident and reflected power waves at the corresponding antennas’ ports, respectively. Then, assuming the same reference impedance at all antennas’ ports (corresponding to the characteristic impedance of the coaxial cable, here equal to 50 Ω), the power waves can be written in terms of the field at the antenna port and the transversal electromagnetic (TEM) coaxial mode as:

(9) $$S_{m,t}=\left\{\begin{array}{cc} {\left.\frac{\langle {\underline{E}}_{m},{\underline{e}}^{\mathrm{TEM}}\rangle }{\langle {\underline{E}}_{t}^{+},{\underline{e}}^{\mathrm{TEM}}\rangle }\right|}_{{\underline{E}}_{m}^{+}=0}, & \mathrm{if}\;m\neq t\\ \frac{\langle {\underline{E}}_{t}-{\underline{E}}_{t}^{+},{\underline{e}}^{\mathrm{TEM}}\rangle }{\langle {\underline{E}}_{t}^{+},{\underline{e}}^{\mathrm{TEM}}\rangle }, & \mathrm{if}\;m=t \end{array}\right.$$

where ${\underline{E}}_{t}$ and ${\underline{E}}_{m}$ are the electric fields at the *t* and *m* coaxial antennas’ ports and ${\underline{E}}_{t}^{+}$ and ${\underline{E}}_{m}^{+}$ the corresponding impressed ones. The coaxial antenna port corresponds to the circular crown surface between the radii $r_{a}$ and $r_{b}$, as shown in Figure 2.

**Figure 3:**
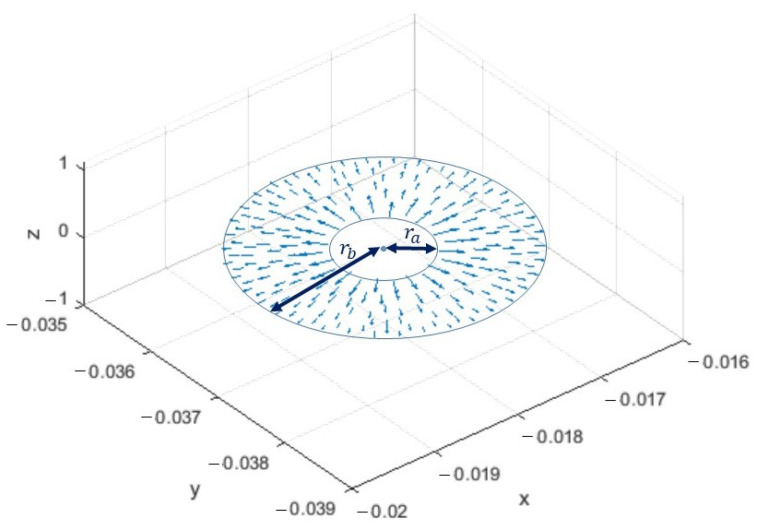
TEM mode on the coaxial port surface.

The vector ${\underline{e}}^{\mathrm{TEM}}$ is the modal function of the coaxial cable TEM mode ([28] Ch. 5), defined as:

(10) $${\underline{e}}^{TEM}=\frac{1}{\sqrt{2\pi \ln(\frac{r_{b}}{r_{a}})}}\frac{\hat{\rho }}{\rho }$$

where $\hat{\rho }$ and ρ are the direction and the amplitude of the radial variable from the center of the coaxial cable in the considered port surface. As shown in Figure 3, the ${\underline{e}}^{TEM}$ mode has a radial behavior with respect to the center of the coaxial on the port surface and decreasing from the smaller circumference to the larger one. Finally, $\langle \cdot \rangle$ in (9) is the inner product between the two considered vectors.

Enforcing a TEM impressed field at the *t* antenna port with amplitude equal to 1 V, ${\underline{E}}_{t}^{+}={\underline{e}}^{\mathrm{TEM}}$ and (9) can be simplified as:

(11) $$S_{m,t}=\left\{\begin{array}{cc} {\left.\langle {\underline{E}}_{m},{\underline{e}}^{\mathrm{TEM}}\rangle \right|}_{{\underline{E}}_{m}^{+}=0}, & \mathrm{if}\;m\neq t\\ \langle {\underline{E}}_{t}-{\underline{E}}_{t}^{+},{\underline{e}}^{\mathrm{TEM}}\rangle , & \mathrm{if}\;m=t \end{array}\right.$$

Then, we can assume that the electric field propagating in a coaxial cable is the TEM field only, writing:

(12) $${\underline{E}}_{m}=V_{m}{\underline{e}}^{\mathrm{TEM}}$$

and, substituting (12) in (11), we obtain that $S_{m,t}=V_{m}$. Hence, applying the same procedure for the background (reference) scenario and the one under test, we obtain that the scattered field at the *m*-th antenna port due to the *t*-th transmitting antenna can be written as:

(13) $${\underline{E}}_{m}^{\mathrm{sct}}=\left(S_{m,t}^{\mathrm{tot}}-S_{m,t}^{\mathrm{inc}}\right){\underline{e}}^{\mathrm{TEM}},$$

where the scattered field is written in terms of the scattering parameters at the antennas’ ports in the background and under test scenarios. The same procedure can be applied for the case of m=t.

## 4. Discretization of the CSI Variables

In this section, we analyze different discretizations of the CSI variables in order to implement numerically, via an FEM approach, the CSI method described in Section 3.

First, the entire volume, Ω, is discretized via tetrahedral cells, where the complex relative permittivity is evaluated in each cell’s barycenter and assumed constant in the cell. This discretization of the complex relative permittivity allows writing the dielectric contrast as:

(14) $$\chi \left(\underline{r}\right)≅\sum_{i=1}^{I}\chi_{i}p_{i}\left(\underline{r}\right),$$

where $\chi_{i}$ corresponds with the dielectric contrast in the *i*-th tetrahedron $C_{i}$, *I* is the number of tetrahedra in the whole domain, and $p_{i}\left(\underline{r}\right)$ is the pulse function, defined as:

(15) $$p_{i}\left(\underline{r}\right)=\left\{\begin{array}{cc} 1 & \underline{r}\in C_{i}\\ 0 & \mathrm{elsewhere}. \end{array}\right.$$

The field radiated by each antenna *t* can be written as a linear combination of a set of vector basis functions, ${\underline{N}}_{e}\left(\underline{r}\right)$, associated with each edge *e* of the tetrahedral mesh; the field basis functions are curl-conforming and have a spatial domain corresponding with all the tetrahedra with the considered edge *e* in common. Moreover, they have a constant tangential component along the associated edge *e* and no tangential component along the other edges of the tetrahedra. This kind of basis function is often exploited in FEM problems with tetrahedral discretization [29]. Then, the fields can be written as:

(16) $$\begin{array}{ccc} {\underline{E}}_{t}^{\mathrm{tot}}\left(\underline{r}\right) & ≅ & \sum_{e=1}^{E}E_{t,e}^{\mathrm{tot}}{\underline{N}}_{e}\left(\underline{r}\right) \end{array}$$

(17) $$\begin{array}{ccc} {\underline{E}}_{t}^{\mathrm{inc}}\left(\underline{r}\right) & ≅ & \sum_{e=1}^{E}E_{t,e}^{\mathrm{inc}}{\underline{N}}_{e}\left(\underline{r}\right) \end{array}$$

(18) $$\begin{array}{ccc} {\underline{E}}_{t}^{\mathrm{sct}}\left(\underline{r}\right) & ≅ & \sum_{e=1}^{E}E_{t,e}^{\mathrm{sct}}{\underline{N}}_{e}\left(\underline{r}\right) \end{array}$$

where *E* is the total number of edges of the tetrahedral mesh, and $E_{t,e}^{\mathrm{tot}}$, $E_{t,e}^{\mathrm{inc}}$, and $E_{t,e}^{\mathrm{sct}}$ are the coefficients of the total, incident, and scattered field, respectively.

### 4.1. Standard Contrast Source Discretization

The first considered discretization of the contrast source variables is the standard one [30]. In this kind of discretization, the contrast sources are associated to each tetrahedron, and this allows to write ${\underline{\omega }}_{t}$ in a similar way as for the dielectric contrast in (14):

(19) $${\underline{\omega }}_{t}\left(\underline{r}\right)≅\sum_{i=1}^{I}{\underline{\omega }}_{t,i}p_{i}\left(\underline{r}\right),$$

where ${\underline{\omega }}_{t,i}$ are the *vector* coefficients and correspond to the value of the contrast source in the barycenter of the *i*-th tetrahedron. These vector coefficients can be explicitly written via (2) as:

(20) $${\underline{\omega }}_{t,i}=\chi \left({\underline{r}}_{i}\right){\underline{E}}_{t}^{\mathrm{tot}}\left({\underline{r}}_{i}\right),$$

where ${\underline{r}}_{i}$ is the position vector at the barycenter of the considered *i*-th tetrahedron. We can notice that, with the discretization in (19), the contrast source is assumed constant in amplitude and direction in each cell, loosing the linear variability instead available in the fields’ discretization (18).

Applying the Galerkin weighted residual testing and substituting (18) and (19) in (4), we obtain the following discretized wave equation:

(21) $$\left([U]-[V]\right)\left[E_{t}^{\mathrm{sct}}\right]=\left[\underline{R}\right]\cdot \left[{\underline{\omega }}_{t}\right],$$

where [U] and [V] are E×E matrices, usually called FEM stiffness and mass matrices, respectively. Each element (i,j) of [U] and [V] can be written as:

(22) $$\begin{array}{ccc} {\left[U\right]}_{i,j} & = & \int_{\Omega }\left(\nabla \times {\underline{N}}_{i}\right)\cdot \left(\nabla \times {\underline{N}}_{j}\right)d^{3}\underline{r}, \end{array}$$

(23) $$\begin{array}{ccc} {\left[V\right]}_{i,j} & = & \int_{\Omega }k_{b}^{2}{\underline{N}}_{i}\cdot {\underline{N}}_{j}d^{3}\underline{r}. \end{array}$$

The integrals in (22) and (23) are known in closed form [29] thanks to the chosen test and basis functions. Moreover, $\left[E_{t}^{\mathrm{sct}}\right]$ is an E×1 array that collects the scattering field coefficients (18), and $\left[{\underline{\omega }}_{t}\right]$ is an I×1 array collecting the *vector* contrast source coefficients (20). Due to its vector nature, each element of the $\left[{\underline{\omega }}_{t}\right]$ array corresponds to three scalar quantities that have to be updated at each CSI iteration. Finally, $\left[\underline{R}\right]$ is a vector matrix with dimension of E×I; each element (i,j) corresponds to:

(24) $${\left[\underline{R}\right]}_{i,j}=\int_{\Omega }k_{b}^{2}{\underline{N}}_{i}\left(\underline{r}\right)p_{j}\left(\underline{r}\right)d^{3}\underline{r}.$$

In (21), the symbol “·” identifies the scalar product between the vector matrix $\left[\underline{R}\right]$ and the vector array $\left[{\underline{\omega }}_{t}\right]$. Due to the use of vector arrays and matrices, the numerical implementation is quite complex and, in particular, requires a dyadic expression for the discretized operators $G_{D}$ and $G_{S}$ in (6) and (7).

### 4.2. Proposed Contrast Source Discretization

Here, we propose a novel discretization of the contrast source variables that avoids the use of vector coefficients and, as a consequence, of vector arrays, matrices, and dyadic operators; moreover, at the same time, a linear variation of the contrast source is kept within each cell, as it is for the fields.

In (16), (17) and (18), each basis function ${\underline{N}}_{e}\left(\underline{r}\right)$ is labeled with the generic edge e=1,…,E, where *E* is the total number of edges in the mesh. The basis function ${\underline{N}}_{e}\left(\underline{r}\right)$ has, as definition domain, the volume of the $Q_{e}$ tetrahedra that have in common the considered edge *e*. Then, we can write a new basis function ${\underline{\tilde{N}}}_{e,q}\left(\underline{r}\right)$ as:

(25) $${\underline{\tilde{N}}}_{e,q}\left(\underline{r}\right)={\underline{N}}_{e}\left(\underline{r}\right)p_{\alpha (e,q)}$$

where the pulse function $p_{\alpha (e,q)}$ is defined in (15), and α(e,q) returns the i-th tetrahedron index as a function of the edge index *e* and the local index $q=1,\ldots ,Q_{e}$ of the tetrahedra that have in common the considered edge.

Exploiting (25), the total field in (16) can be rewritten as:

(26) $${\underline{E}}_{t}^{\mathrm{tot}}\left(\underline{r}\right)≅\sum_{e=1}^{E}E_{t,e}^{\mathrm{tot}}\sum_{q=1}^{Q_{e}}{\underline{N}}_{e}\left(\underline{r}\right)p_{\alpha (e,q)}=\sum_{e=1}^{E}\sum_{q=1}^{Q_{e}}E_{t,e}^{\mathrm{tot}}{\underline{\tilde{N}}}_{e,q}\left(\underline{r}\right)$$

Substituting (14) and (26) into (2), we obtain:

(27) $$\begin{array}{ccc} {\underline{\omega }}_{t}\left(\underline{r}\right) & ≅ & \left[\sum_{i=1}^{I}\chi_{i}p_{i}\left(\underline{r}\right)][\sum_{e=1}^{E}\sum_{q=1}^{Q_{e}}E_{t,e}^{\mathrm{tot}}{\underline{\tilde{N}}}_{e,q}\left(\underline{r}\right)\right]\\ & = & \sum_{e=1}^{E}E_{t,e}^{\mathrm{tot}}\sum_{i=1}^{I}\sum_{q=1}^{Q_{e}}\chi_{i}p_{i}\left(\underline{r}\right){\underline{\tilde{N}}}_{e,q}\left(\underline{r}\right)\\ & = & \sum_{e=1}^{E}\sum_{q=1}^{Q_{e}}[E_{t,e}^{\mathrm{tot}}\chi_{\alpha (e,q)}]p_{\alpha (e,q)}\left(\underline{r}\right){\underline{\tilde{N}}}_{e,q}\left(\underline{r}\right)\\ & = & \sum_{e=1}^{E}\sum_{q=1}^{Q_{e}}\omega_{t,e,\alpha (e,q)}{\underline{\tilde{N}}}_{e,q}\left(\underline{r}\right). \end{array}$$

**Figure 4:**
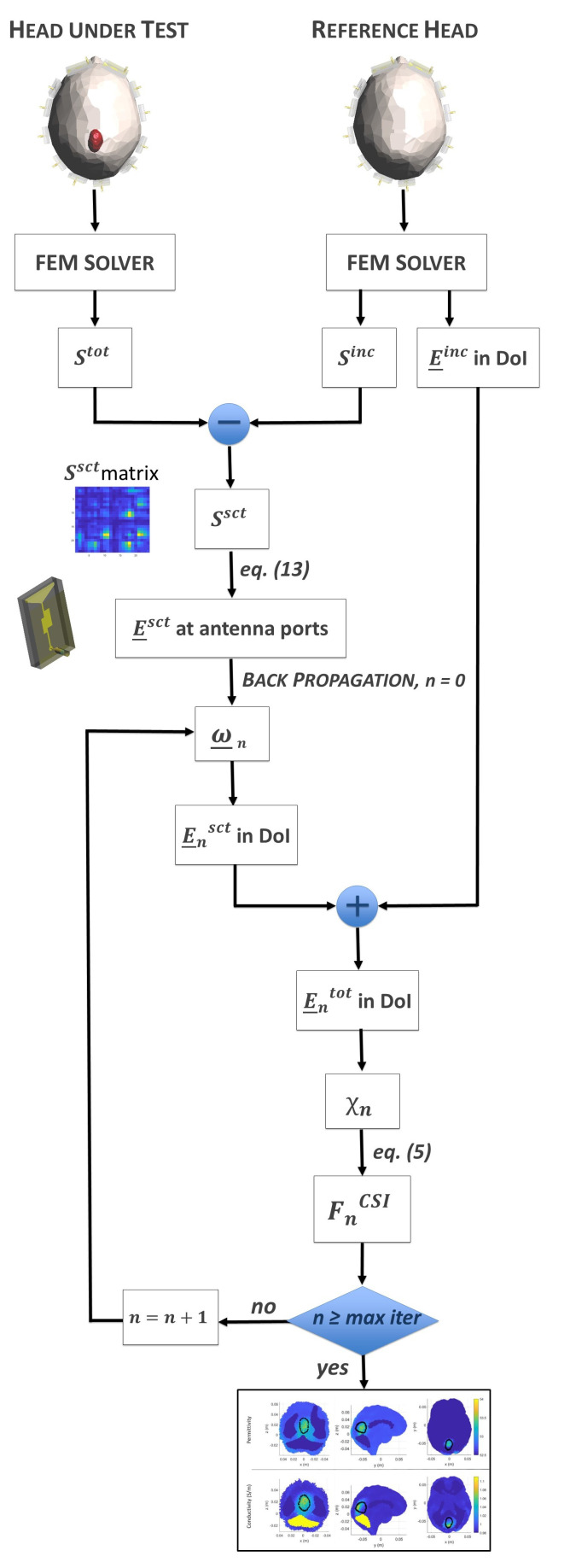
Flowchart of the implemented CSI algorithm.

In (27), during the steps to reach the final discretization, the pulse function $p_{i}\left(\underline{r}\right)$ is left out because the domain of ${\underline{\tilde{N}}}_{e,q}\left(\underline{r}\right)$ already bounds within each tetrahedron. In this discretization, the contrast sources are now written as a linear combination of *vector* basis functions weighted by *scalar* coefficients. Moreover, they use the same vector basis functions of the field, allowing a linear variation within each cell. Substituting (18) and (27) in (4), and applying the Galerkin weighted residual testing, we obtain the following discretized wave equation:

(28) $$\left([U]-[V]\right)\left[E_{t}^{\mathrm{sct}}\right]=\left[R\right]\left[\omega_{t}\right],$$

where, unlike (21), the right-hand side is the standard matrix/vector product between scalar quantities. Indeed, $\left[\omega_{t}\right]$ is a 6I array (i.e., the contribution of the 6 edges for each element *i*) that contains the scalar coefficients of (27), and [R] is a matrix with dimension E×6I with each element equal to:

(29) $${\left[R\right]}_{m,n}=\int_{\Omega }k_{b}^{2}{\underline{N}}_{m}\left(\underline{r}\right)\cdot {\underline{\tilde{N}}}_{n}\left(\underline{r}\right)d^{3}\underline{r},$$

where ${\underline{\tilde{N}}}_{n}\left(\underline{r}\right)$ has the index *n* that coincides with the double indexing (e,q) in (27). To better clarify the overall procedure, in Figure 4, we show a flowchart of the implemented CSI algorithm.

The main advantage of the proposed discretization is that the field and the contrast sources are written with the same basis functions, reducing the update procedure just to the scalar coefficients. This characteristic facilitates the CSI algorithm implementation, avoiding vector arrays and matrices as well as dyadic operators. Moreover, it improves the discretization accuracy, as detailed in Section 5.

## 5. Numerical Results

**Figure 5:**
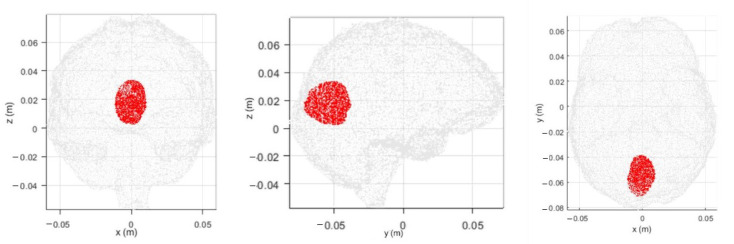
Views of the considered hemorrhagic stroke (in red) within the brain.

In this section, we report the numerical comparison, in terms of accuracy in the discretized wave equation, between the standard and the novel discretizations of the contrast sources in a homogeneous head (see Section 5.1). Then, the performances of the two types of discretization are analyzed and compared in the 3D imaging of a hemorrhagic stroke inside the multitissue head shown in Figure 1 (see Section 5.2). All the simulations are performed at 1 GHz. The stroke has the permittivity and conductivity of the blood, i.e., $\epsilon_{r}=64.41$ and σ=1.58 S/m [26]; it is positioned in the gray matter and has a realistic shape, which was obtained by medical images [31], as shown in Figure 5. The domain is discretized via tetrahedral cells with an edge dimension of 3 mm, corresponding to around λ/15 if we consider a background medium equal to the average of the dielectric properties of all the head tissues, $\epsilon_{mean}=45.37$ and $\sigma_{mean}$ = 0.77 S/m [32]. For each radiating antenna *t*, the field distribution in the domain is evaluated through the EM solver considering the absorbing boundary condition on the external surface of the whole domain ([28] Ch. 3).

### 5.1. Comparison between the Contrast Source Discretizations

Initially, a homogeneous head phantom is exploited with dielectric properties corresponding to the average of the dielectric properties of all the tissues inside the head. The first step for the comparison is the assessment of the accuracy in the discretized wave equations for the two different contrast source discretizations. In particular, we evaluate the difference between the left-hand side (LHS) and the right-hand side (RHS) in (21) and (28), using as input the same scattered and total fields (obtained in the considered scenario via the FEM solver), as well the same (corresponding) dielectric contrast. In order to have quantitative indices of the errors between LHS and RHS in the two contrast source discretizations, we evaluate the Euclidean norm, η, and the relative one, $\eta_{r}$, as

(30) $$\eta =\sqrt{\sum_{e=1}^{E}{\left|{\left[LHS\right]}_{e}-{\left[RHS\right]}_{e}\right|}^{2}}$$

(31) $$\eta_{r}=\frac{\eta }{\sqrt{\sum_{e=1}^{E}{\left|{\left[LHS\right]}_{e}\right|}^{2}}}.$$

where ${\left[LHS\right]}_{e}$ and ${\left[RHS\right]}_{e}$ are the *e*-th elements of the LHS and RHS arrays, respectively. The results for (30) and (31) are reported in Table 1.

The higher error between the LHS and the RHS obtained with the standard discretization is due to the assumption in (20) that the field value and direction are considered constant within each tetrahedron. Therefore, we evaluate the errors in the scattered field coefficients, $\left[E^{\mathrm{sct}}\right]$, that are obtained through (21) and (28), comparing them with the scattered field obtained with the FEM solver as the difference between the total and incident fields. The errors are reported in Table 2; a significantly lower error is obtained with the proposed contrast source discretization.

Finally, in order to estimate the accuracy of the proposed discretization related with the overall CSI procedure, we evaluate the minimum cost functional value of the algorithm for the two discretizations. In particular, we substitute the exact values of the dielectric contrast and the contrast sources in the cost functional (5). In Table 3, the results for the two discretizations, separately for $F^{S}$ and $F^{D}$, are reported.

The novel discretization has a minimum value of $F^{CSI}$ of around 10 orders of magnitude lower than in the standard discretization.

### 5.2. Brain Stroke Imaging

In this section, there is the analysis of the CSI algorithm performances, applying both the standard and the proposed contrast source discretizations. In particular, the standard discretization is implemented by substituting $\underline{\tilde{N}}\left(\underline{r}\right)$ with $\underline{\tilde{N}}\left({\underline{r}}_{i}\right)$, where ${\underline{r}}_{i}$ is the barycenter position vector of the tetrahedron where $\underline{\tilde{N}}\left(\underline{r}\right)$ is defined. In this way, the proposed discretization is equivalent to the standard one, with the contrast source variables described by scalar coefficients and the direction variability within each tetrahedron guaranteed by the contribution of the six different (now constant) vector basis functions defined in it (one for each tetrahedron edge). Hence, for all the comparisons reported in the following, we are able to use the same code implementation in the same conditions, just with a different discretization of the contrast source variables.

The considered scenario comprises the multitissue head phantom, described in Section 2, in order to identify the location and dielectric characteristics of the hemorrhagic stroke, shown in Figure 5. The scattering parameters at the antennas’ ports are simulated with a tetrahedral mesh different from the one used to generate the incident field and the operators in the CSI algorithm. This choice allows to avoid inverse crime and to assess the robustness of the proposed method with respect to inaccuracies in the input data. The CSI algorithm starts with the initial guess computation. As described in [30], the initial guess cannot be equal to zero because the cost functional is undefined with a zero contrast source. The initial guess corresponds to the contrast source variable that minimizes the data-error equation in the cost functional $F^{S}\left\{{\underline{\omega }}_{t,n}\left(\underline{r}\right)\right\}$. This minimization is obtained via backpropagation [9]. In [33], there is some recent research for a new analytical method, which provides good initial guesses.

**Figure 6:**
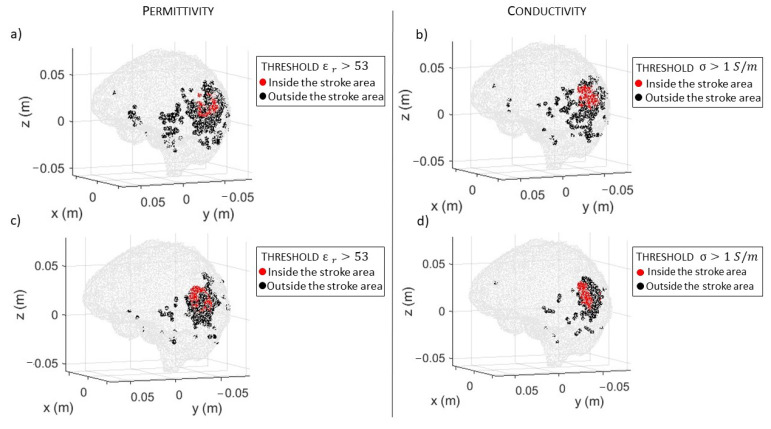
CSI algorithm results with the standard (**a**,**b**) and the proposed discretization (**c**,**d**). The spots correspond to the volume of the stroke identified by the algorithm after 100 iterations: permittivity (**a**,**c**) and conductivity (**b**,**d**). Red inside and black outside the stroke area.

**Figure 7:**
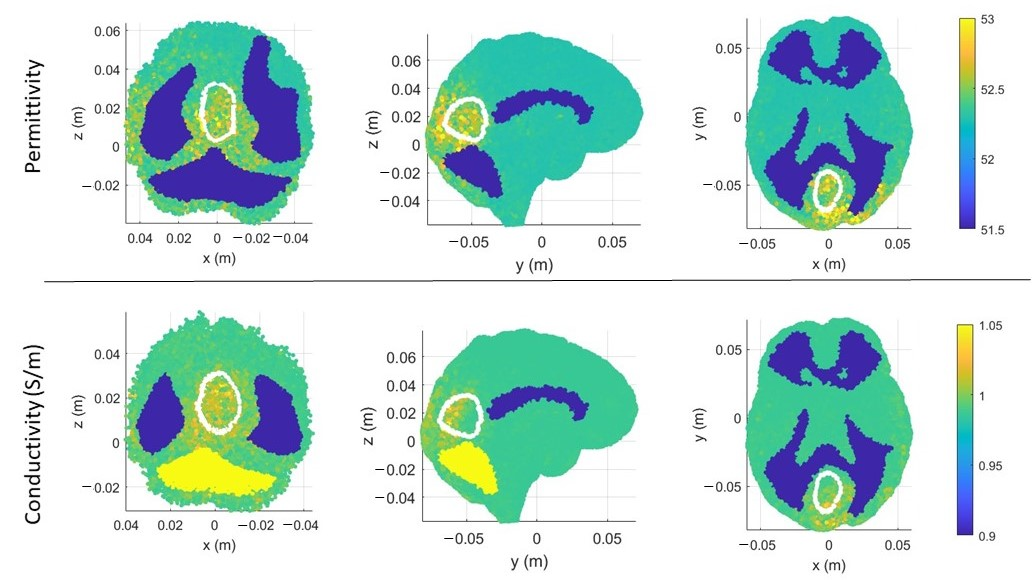
CSI algorithm results with the standard discretization. Shape, position, and dielectric property values of the stroke identified by the CSI algorithm after 100 iterations. On the first row, three different views of the permittivity, and on the second row, three different views of the conductivity. The perimeter of the expected stroke shape is shown in white.

**Figure 8:**
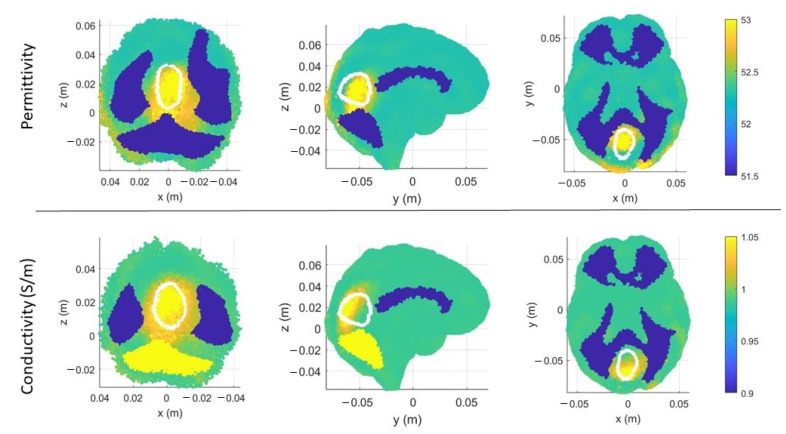
CSI algorithm results with the proposed discretization. Shape, position, and dielectric properties values of the stroke identified by the CSI algorithm after 100 iterations. On the first row, three different views of the permittivity, and on the second row, three different views of the conductivity. The perimeter of the expected stroke shape is shown in white.

In Figure 6a,b, there are the volumes of the stroke permittivity and conductivity identified by the CSI algorithm after 100 iterations with the standard discretization. In this figure, each dot is the barycenter of each cell with $\epsilon_{r}>53$ (Figure 6a) and σ > 1 S/m (Figure 6b). The color denotes if the dot is inside (red) or outside (black) the expected stroke region. Instead, in Figure 7, there are the stroke reconstructed dielectric properties (after 100 iterations) in the three main cuts of the brain; the perimeter of the expected stroke shape is also shown in white. In Figure 7, it is possible to see a slightly lighter halo in the region of the stroke, but the algorithm struggles to reconstruct the target. Then, the same graphs are reported using the proposed discretization in Figure 6c,d and Figure 8. In this case, the shape of the stroke is better focused, and the values of both permittivity and conductivity distribution are higher in the stroke region.

**Figure 9:**
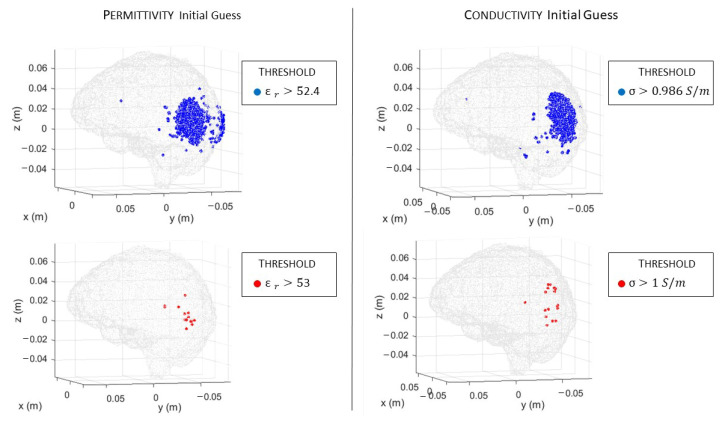
CSI algorithm initial guess with the proposed discretization. On the first row, in blue, the region of the stroke identified by the algorithm, and on the second row, in red, the spots with higher dielectric properties values: on the left, permittivity, and on the right, conductivity.

Then, we exploit the information given by the initial guess in order to improve the algorithm convergence and the simulation time with the proposed discretization. In particular, as reported in Figure 9, the initial guess, evaluated through backpropagation, is able to detect the region of the brain affected by the stroke, clearly identified through the thresholds $\epsilon_{r}>52.4$ (Figure 9, first row, left) and σ > 0.986 S/m (Figure 9, first row, right). In Figure 9 (second row), we also reported the same thresholds used in Figure 6, but in this case, they detect just a few spots. Then, during the iterations, the CSI method improves the dielectric properties values and the shape of the stroke.

**Figure 10:**
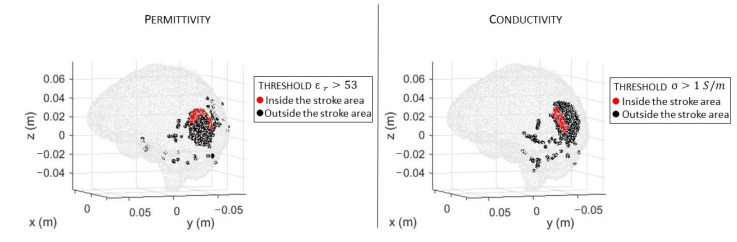
CSI algorithm results with the proposed discretization, considering half-brain DoI. The spots corresponds to the volume of the stroke identified by the algorithm after 100 iterations: on the left, permittivity, and on the right, conductivity. Red inside and black outside the stroke area.

**Figure 11:**
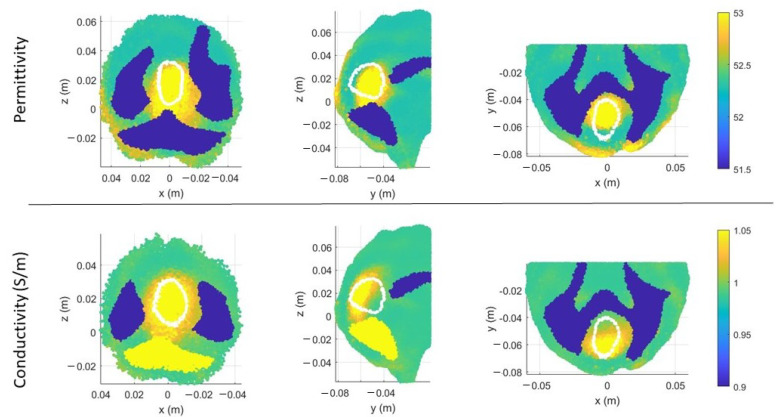
CSI algorithm results with the proposed discretization, considering half-brain DoI. Shape, position, and dielectric properties values of the stroke identified by the CSI algorithm after 100 iterations. On the first row, three different views of the permittivity, and on the second row, three different views of the conductivity. The perimeter of the expected stroke shape is shown in white.

**Figure 12:**
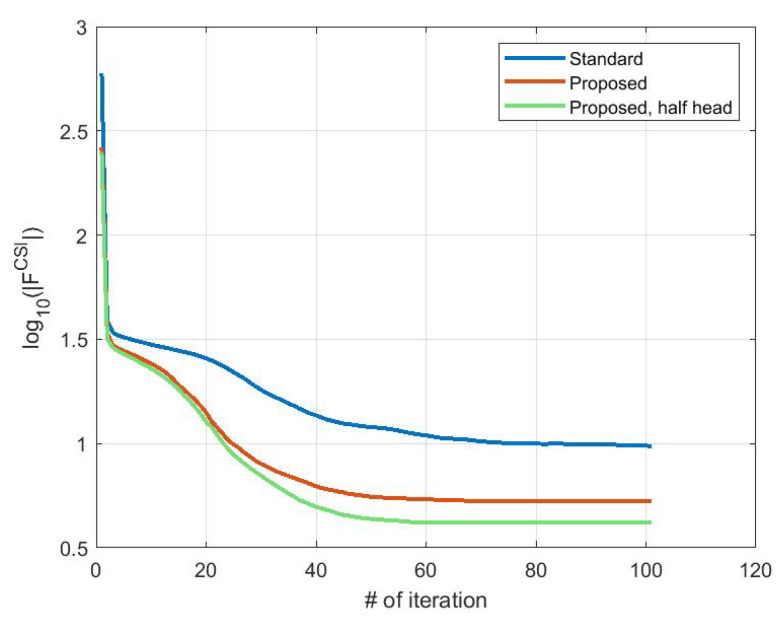
Cost functional in (5) during the iterations in a logarithmic scale: standard discretization (blue line), proposed discretization (red line), and proposed discretization with half head (green line).

This information allows limiting the DoI, reducing the number of unknowns significantly and, consequently, the simulation time: a reduction to 60% of the initial unknowns leads to gaining around 30% of the simulation time, without considering the solution of the system that is equal in both cases. In particular, we limit the DoI to the half-back part of the brain. Moreover, for this case, we report the results after 100 iterations. In Figure 10, there is the estimated stroke volume and, in Figure 11, the corresponding permittivity and conductivity. Comparing the stroke reconstructions in the three tests, the proposed discretization obtains a better focus on the stroke and higher values of the dielectric properties in the stroke region. In fact, considering just the stroke region elements, the percentage with $\epsilon_{r}>53.00$ is 2.00% in the standard discretization, 28.41% in the proposed discretization, and 51.30% in the proposed discretization with half head. Moreover, Figure 12 shows the cost functional in (5) in logarithmic scale during the CSI algorithm convergence for the three tests. As expected, the cost functional in the proposed discretization (red line) starts and reaches the convergence with lower values with respect to the standard discretization (blue line), and a further improvement is obtained using the proposed discretization with half head (green line).

Finally, Table 4 reports the obtained permittivity and conductivity, averaged in the stroke region, and the corresponding standard deviation for all the tests. However, due to the limited range of permittivity values found by the CSI algorithm, these average values are close to each other.

Some recent works on brain microwave imaging through quantitative reconstruction are [10,11], where the distorted Born iterative algorithm is used for the reconstruction of the head dielectric properties. Our results are comparable with those, especially for the focusing and positioning of the reconstructed target. However, an intrinsic limitation of the CSI method [34] is the difficulty in high dielectric contrast reconstruction; indeed, here, the values of the dielectric properties are lower than the expected ones.

## 6. Conclusions and Perspectives

In this paper, a new discretization of the contrast source variables is presented and analyzed in the FEM-CSI algorithm. It is based on the use of the field vector basis functions as well as the contrast source variables. Hence, the field and the contrast sources are written with the same base of vector functions, allowing to update, at each CSI iteration, just scalar coefficients of the contrast sources. This simplifies the implementation of the algorithm, avoiding the use of vector matrices, arrays, and dyadic operators. Moreover, these proposed variables’ discretization leads to a higher accuracy compared with the standard one, thanks to the lower discretization error. The implemented FEM-CSI algorithm, based on the proposed discretization, was validated and compared with the standard one in a 3D multitissue head in order to assess their performances in the same realistic scenario. The head images obtained with the new discretization showed an improvement in stroke identification and reconstruction, which appears better focused.

The future steps of this work deal with collecting a priori information about the distribution of the head tissues, e.g., [35], where the segmented images obtained through ultrasounds are used as numerical background in the CSI algorithm for the MWI of breast cancer. In addition, the CSI performances can be optimized through a regularization procedure, as in [27]. Moreover, the algorithm implementation can be sped up via programmable system-on-chip solutions [36] or via graphic processing units [11]. Finally, we plan to apply the implemented FEM-CSI algorithm to experimental data obtained through the system described in [20], applying a proper calibration technique, such as the one described in [37], to mitigate the discrepancy between the simulated and real antennas due to manufacturing tolerances.

## Figures and Tables

**Table 1:** Error between RHS and LHS.

	η	$\eta_{r}$
Standard	$9.52\times 10^{-8}$	$2.03\times 10^{-5}$
Novel	$7.47\times 10^{-11}$	$1.59\times 10^{-8}$

**Table 2:** $\left[E^{\mathrm{sct}}\right]$ error.

	η	$\eta_{r}$
Standard	$4.13\times 10^{-4}$	$2.03\times 10^{-5}$
Novel	$5.14\times 10^{-10}$	$2.52\times 10^{-11}$

**Table 3:** Cost functional.

	$F^{S}$	$F^{D}$
Standard	$9.87\times 10^{-9}$	$9.51\times 10^{-10}$
Novel	$6.48\times 10^{-20}$	$1.11\times 10^{-23}$

**Table 4:** Stroke Dielectric Properties.

	Exact	Standard Discr.	Proposed Discr.	Proposed Discr. Half brain
Permittivity	63.41	52.53±0.18 max = 54.24	52.80±0.31 max = 53.69	53.00±0.42 max = 54.24
Conductivity (S/m)	1.58	1.00±0.01 max = 1.15	1.02±0.02 max = 1.18	1.03±0.03 max = 1.19

## Abbreviations

The following abbreviations are used in this manuscript:

CSI	Contrast Source Inversion
MWI	Microwave Imaging
DoI	Domain of Interest
FEM	Finite Element Method
TEM	Transversal Electromagnetic
LHS	Left-Hand Side
RHS	Right-Hand Side
RMSE	Root Mean Square Error
